# The advanced lung cancer inflammation index is associated with mortality in peritoneal dialysis patients

**DOI:** 10.1186/s12882-024-03645-4

**Published:** 2024-06-25

**Authors:** Zhouhao Ren, Jiaying Wu, Shaorui Wu, Mengwei Zhang, Shuijuan Shen

**Affiliations:** 1 Department of Nephrology, Shaoxing People′s Hospital, Shaoxing, 312000 China; 2 Department of Nephrology, Taizhou Central Hospital (Taizhou University Hospital), Taizhou, 318000 China; 3 Shaoxing University School of Medicine, Shaoxing, 312000 China

**Keywords:** Advanced lung cancer inflammation index, Peritoneal dialysis, All-cause mortality, Cardiovascular mortality

## Abstract

**Background:**

There is still a very high morbidity and mortality rate for patients undergoing peritoneal dialysis (PD). The advanced lung cancer inflammation index (ALI) has been demonstrated to be associated with the prognosis in multiple types of cancers. Like in cancer, systemic chronic low-grade inflammation is one of the distinguishing features of PD patients. Therefore, we aimed to investigate the relationships between the ALI and all-cause and cardiovascular disease (CVD) mortality in PD patients.

**Methods:**

Patients who started PD at Shaoxing People’s Hospital between 1 January 2013 and 31 December 2020 (*n* = 277) were recruited and followed up until 1 July 2023. They were divided into high-ALI group and low-ALI group according to the median of ALI. Kaplan–Meier curves and multivariate Cox regression analyses were used to assess the associations between the ALI and all-cause and CVD mortality. Receiver operating characteristic (ROC) curves were constructed, and the area under the curve (AUC) was calculated to determine the predictive power of the ALI for all- cause and CVD mortality.

**Results:**

During the median follow-up of 40.50 months (interquartile range, 26.42–59.77 months), a total of 55 patients died, 31 of whom died due to CVD. Kaplan–Meier curves revealed that patients in the low-ALI group had significantly lower cumulative and cardiovascular cumulative survival rates than did those in the high-ALI group (all *P* < 0.001). After we corrected for confounders, the risk of all-cause and CVD mortality was significantly greater in the low-ALI group than in the high-ALI group [hazard ratio (HR) 1.944, 95% confidence interval (CI) 1.068–3.540, *P* = 0.030, and HR 2.672, 95% CI 1.188–6.009, *P* = 0.017, respectively]. The predictive value of ALI (AUC = 0.708, 95% CI 0.630–0.786, *P* < 0.001) for all-cause mortality was superior to albumin (AUC = 0.644, 95% CI 0.556–0.726, *P* < 0.001), body mass index (AUC = 0.581, 95% CI 0.496–0.659, *P* = 0.069) and neutrophil-to-lymphocyte ratio (AUC = 0.675, 95% CI 0.596–0.754, *P* < 0.001).

**Conclusion:**

A lower ALI is an independent risk factor for all-cause and cardiovascular mortality in PD patients. The ALI may be an effective indicator for predicting outcomes in PD patients.

**Supplementary Information:**

The online version contains supplementary material available at 10.1186/s12882-024-03645-4.

## Background

Peritoneal dialysis (PD) is an effective treatment for patients with end-stage renal disease. Although studies have reported that PD improves the survival and quality of life of patients, the mortality of PD patients remains high. More than half of the deaths of PD patients are related to cardiovascular disease (CVD) [1]. Chronic systemic inflammation is common in PD patients, and approximately 12-65% of PD patients worldwide experience inflammation [2]. There is mounting evidence that chronic inflammation is one of the major risk factors for poor prognosis in PD patients. Chronic inflammation leads to protein-energy wasting (PEW) and is associated with poor cardiovascular outcomes [3–5]. Investigating any treatable cause of inflammation in PD patients with persistently elevated C-reactive protein levels and continuously monitoring nutritional status to identify PD patients with PEW has become one of the key measures for reducing cardiovascular risk [6].

The advanced lung cancer inflammation index (ALI), which is based on albumin (ALB), body mass index (BMI), and the neutrophil-to-lymphocyte ratio (NLR), was first developed by Jafri et al. and has been proven to be a novel inflammatory parameter for predicting the prognosis of patients with metastatic non-small cell lung cancer [7]. It has also been associated with outcomes in chronic inflammatory diseases such as other tumours, heart failure and hypertension [8–11]. However, to our knowledge, no study has evaluated the prognostic value of the ALI in PD patients. In this retrospective study, we aimed to evaluate the associations of the ALI with all-cause and cardiovascular mortality in PD patients.

## Methods

### Study design and participant selection

**Fig. 1:**
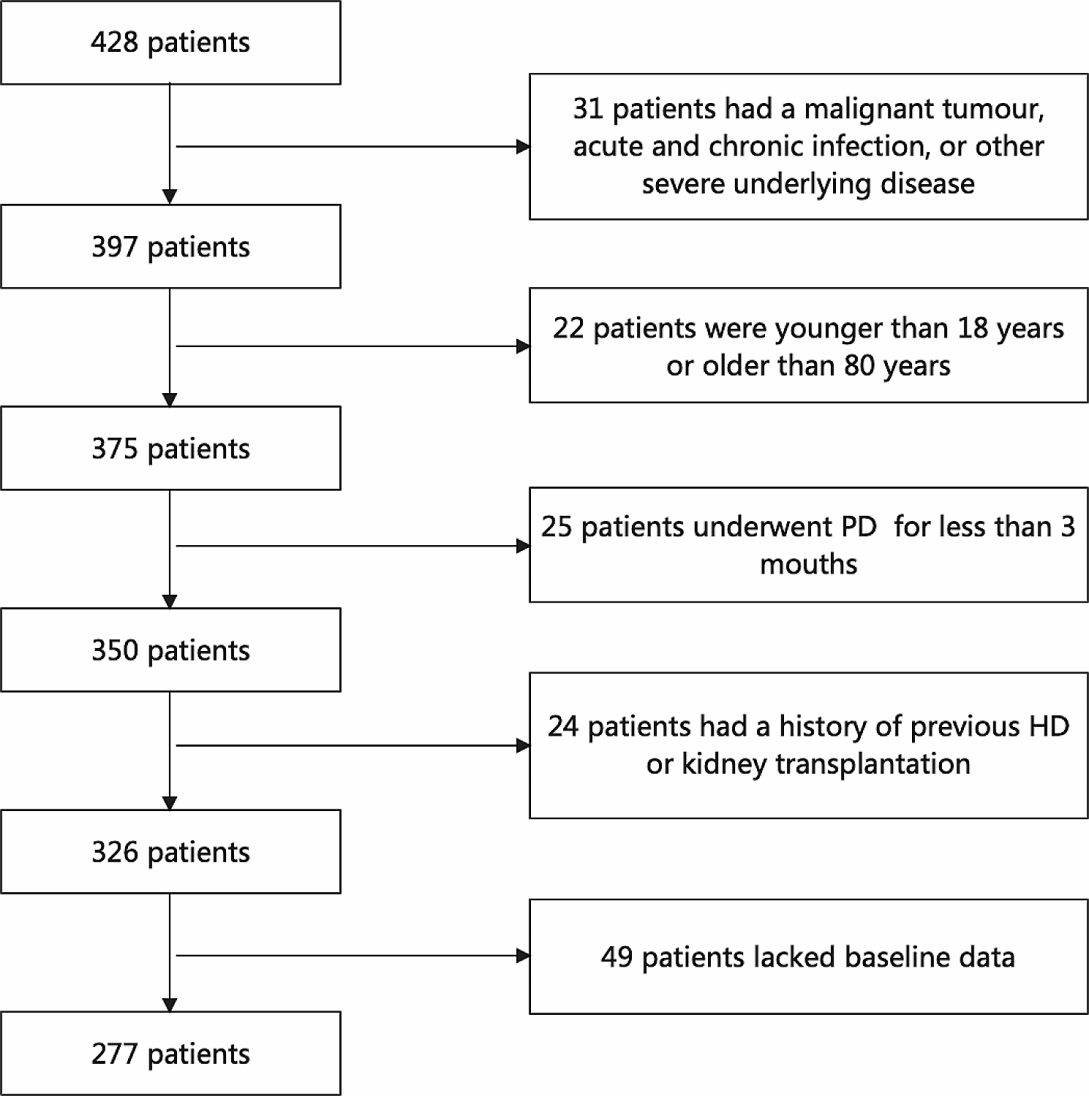
Flow chart of patient enrolment and exclusion in this study

This study included patients who started PD treatment between 1 January 2013 and 31 December 2020 at Shaoxing People′s Hospital. The inclusion criteria were PD patients who have catheters placed at our hospital, underwent regular follow-ups for more than 3 months, and were over 18 years old. The exclusion criteria were as follows: (1) had a malignant tumour, acute infection (pneumonia, bronchiolitis, urinary tract infection, etc.), chronic infection (tuberculosis, active hepatitis, etc.), or other severe underlying disease (chronic obstructive pulmonary disease, cirrhosis, etc.); (2) had a history of previous haemodialysis (HD) or kidney transplantation; (3) were aged < 18 years or > 80 years; (4) underwent PD for less than 3 months; and (5) lacked baseline data. The enrolled patients received 1.5% or 2.5% lactate-buffered glucose-based PD solutions (Dianeal®, Baxter, China). The dialysis modality was continuous ambulatory peritoneal dialysis (CAPD) with 2–4 exchanges per with 1.5–2 L of dwell volume. During dialysis, the dialysis regimen would be promptly adjusted based on ultrafiltration, blood pressure, edema, and other factors to ensure that patients receive adequate dialysis. Finally, 277 patients were enrolled in this study (Fig. 1). The baseline characteristics were collected in the first 1–3 months after starting PD. This study was conducted in accordance with the ethical standards of the Helsinki Declaration and was approved by the Human Ethics Committees of Shaoxing People′s Hospital (2022-K-Y-128-01). All patients signed informed consent forms before study enrolment.

### Data collection

Baseline demographic data, including sex, age, history of diabetes mellitus, CVD, smoking status, and alcohol consumption, were collected. Clinical and biochemical data included BMI, blood pressure, medication use, white blood cell (WBC) count, neutrophil count, lymphocyte count, haemoglobin (Hb) count, platelet count, high sensitivity C-reactive protein (hs-CRP), ALB, serum creatinine (Scr), serum uric acid (SUA), total cholesterol (TC), triglyceride (TG), high-density lipoprotein cholesterol (HDL-C) and low-density lipoprotein cholesterol (LDL-C). Indicators of dialysis adequacy were as follows: total urea clearance index (Kt/V), creatinine clearance rate (CCR), and 4-h dialysate-to-plasma ratio of creatinine (4-h D/Pcr). The estimated glomerular filtration rate (eGFR) was calculated using the Chronic Kidney Disease Epidemiology Collaboration equation.

### Calculation of the ALI

ALI was calculated as the product of BMI (kg/m^2^) and ALB (g/L) divided by the NLR. NLR was calculated as neutrophil counts divided by lymphocyte counts and BMI was calculated as weight (kg) divided by the square of height (m).

### Follow-up and endpoint definitions

All patients were followed up until death, conversion to HD or kidney transplantation, or 1 July 2023. The primary outcome was all-cause mortality. The secondary outcome was cardiovascular mortality, which was defined as death caused by heart failure, acute myocardial infarction, cardiac arrhythmia, coronary arteriosclerotic heart disease, cardiomyopathy, congenital cardiovascular diseases, valvular heart disease, cardiac arrest, ischaemic stroke, or peripheral vascular disease.

### Statistical analyses

Eligible patients were divided into two groups according to the median values of ALI: a low-ALI group (ALI < 293.07) and high-ALI group (ALI ≥ 293.07). Continuous variables are presented as the mean ± standard deviation (mean ± SD) or median (interquartile range), and an independent-samples t test or Mann–Whitney U test was used to determine the differences between two groups. Categorical variables are presented as percentages and frequencies, and the χ2 test was used to determine the differences between two groups. Kaplan–Meier curves and log-rank tests were used to estimate differences in cumulative survival rates between the two groups. Multivariate Cox regression analyses were performed to evaluate the association between the ALI and mortality. For each outcome of interest, we fitted a series of hierarchically adjusted models: Model 1 was only adjusted for age; Model 2 included Model 1 and further adjusted for demographic data (sex, diabetes mellitus status, CVD status, smoking status, alcohol consumption status, systolic blood pressure, diastolic blood pressure and BMI); Model 3 included Model 2 and further adjusted for medications (angiotensin-converting enzyme inhibitors [ACEIs], angiotensin receptor blockers [ARBs], β-blockers and calcium channel blockers); Model 4 included Model 3 and further adjusted for laboratory markers (Hb, ALB, hs-CRP, SUA, fasting blood glucose, TC, TG, HDL-C, and LDL-C) and 4-h D/Pcr. The dose–response relationship between the ALI and mortality was assessed by restricted cubic splines (RCSs). We used 4 knots located at the 25th, 50th, 75th, and 95th percentiles of the ALI. Receiver operating characteristic (ROC) curve analysis was performed, and the area under the curve (AUC) was calculated to determine the predictive power of the ALI for all- cause and CVD mortality. The statistical analysis was completed with SPSS 26.0 (SPSS, Chicago, IL, USA) and R software (version R-4.3.1, www.project.org). All tests were two-tailed, and *P* < 0.05 was considered to indicate statistical significance.

## Results

### Baseline characteristics

A total of 277 PD patients, 147 males (53.1%) and 130 females (46.9%), participated in this study (Table 1). The mean age was 55.5 ± 13.2 years. The between-group differences in age, diabetes mellitus status, CVD status, BMI, Hb, ALB, hs-CRP, NLR, TG, prealbumin and 4-h D/Pcr were statistically significant (all *P* < 0.05). The other indicators were not significantly different (all *P* > 0.05). The baseline characteristics of all patients are shown in Table 1.

**Table 1:** Comparison of baseline data between two groups of patients grouped according to the median ALI

Variables	All (*n* = 277)	Low ALI (*n* = 138)	High ALI (*n* = 139)	*P* value
Age (year)	55.52 ± 13.20	58.60 ± 12.40	52.47 ± 13.31	< 0.001
Male (n, %)	147 (53.1%)	78 (56.5%)	69 (49.6%)	0.251
Diabetes mellitus (n, %)	83 (30.0%)	50 (36.2%)	33 (23.7%)	0.023
CVD (n, %)	79 (28.5%)	47 (34.1%)	32 (23.0%)	0.042
Smoking (n, %)	68 (24.5%)	32 (23.2%)	36 (25.9%)	0.600
Excessive alcohol consumption (n, %)	39 (14.1%)	19 (13.8%)	20 (14.4%)	0.882
RASIs (n, %)	50 (18.1%)	30 (21.7%)	20 (14.4%)	0.112
CCBs (n, %)	166 (59.9%)	85 (61.6%)	81 (58.3%)	0.573
β-blockers (n, %)	52 (18.8%)	29 (21.0%)	23 (16.5%)	0.341
Body mass index (kg/m^2^)	23.51 ± 3.63	22.40 ± 3.26	24.62 ± 3.65	< 0.001
Systolic blood pressure (mmHg)	134.88 ± 13.58	135.20 ± 14.32	134.57 ± 12.84	0.701
Diastolic blood pressure (mmHg)	81.08 ± 8.88	80.60 ± 9.14	81.56 ± 8.63	0.369
White blood cell count (×10^9^g/L)	5.86 (4.94, 6.87)	5.79 (4.92, 7.01)	5.87 (4.99, 6.83)	0.851
Haemoglobin (g/L)	102.41 ± 13.63	100.57 ± 13.40	104.22 ± 13.66	0.026
Platelet (×10^9^g/L)	202.97 ± 58.43	199.31 ± 64.35	206.61 ± 51.87	0.300
Albumin (g/L)	31.47 ± 4.62	29.23 ± 4.08	33.69 ± 4.03	< 0.001
Hs-CRP (mg/L)	2.09 (0.66, 5.99)	3.08 (1.03, 9.12)	1.48 (0.56, 4.02)	< 0.001
NLR	2.63 (2.07, 3.23)	3.19 (2.69, 3.88)	2.12 (1.77, 2.56)	< 0.001
Blood urea nitrogen (mmol/L)	17.95 ± 5.24	17.82 ± 5.40	18.08 ± 5.09	0.680
Serum creatinine (µmol/L)	504.48 (398.50, 652.48)	493.09 (393.50, 653.01)	512.27 (401.70, 655.40)	0.757
Serum uric acid (µmol/L)	393.62 ± 88.33	386.44 ± 86.11	400.74 ± 90.22	0.178
Total cholesterol (mmol/L)	4.85 (3.98, 5.89)	4.86 (4.16, 5.91)	4.82 (3.88, 5.88)	0.374
Triglyceride (mmol/L)	1.50 (1.07, 2.17)	1.39 (1.01, 1.96)	1.61 (1.16, 2.34)	0.021
HDL-C (mmol/L)	1.12 (0.93, 1.40)	1.13 (0.95, 1.40)	1.11 (0.88, 1.37)	0.400
LDL-C (mmol/L)	3.05 (2.40, 3.88)	3.14 (2.42, 3.86)	2.96 (2.37, 3.91)	0.306
Prealbumin (mg/L)	326.75 ± 80.05	306.68 ± 85.40	346.67 ± 69.08	< 0.001
Fasting blood glucose (mmol/L)	4.83 (4.48, 5.49)	4.82 (4.47, 5.87)	4.83 (4.49, 5.34)	0.644
Kt/V (per week)	2.43 (2.10, 2.93)	2.44 (2.04, 2.87)	2.43 (2.11, 2.97)	0.631
CCR (per week)	72.61 (29.52, 100.44)	75.08 (34.75, 102.27)	70.91 (27.73, 97.03)	0.617
4-h D/Pcr	0.69 ± 0.12	0.72 ± 0.12	0.66 ± 0.12	< 0.001

ALI advanced lung cancer inflammation index, CVD cardiovascular disease, RASIs renin-angiotensin system inhibitors, CCBs calcium channel blockers, Hs-CRP high sensitivity C-reactive protein, NLR neutrophil-to-lymphocyte ratio, HDL-C high-density lipoprotein cholesterol, LDL-C low-density lipoprotein cholesterol, Kt/V total urea clearance index, CCR creatinine clearance rate, 4-h D/Pcr 4-h dialysate-to-plasma ratio of creatinine

### Relationship between the ALI and mortality

**Fig. 2:**
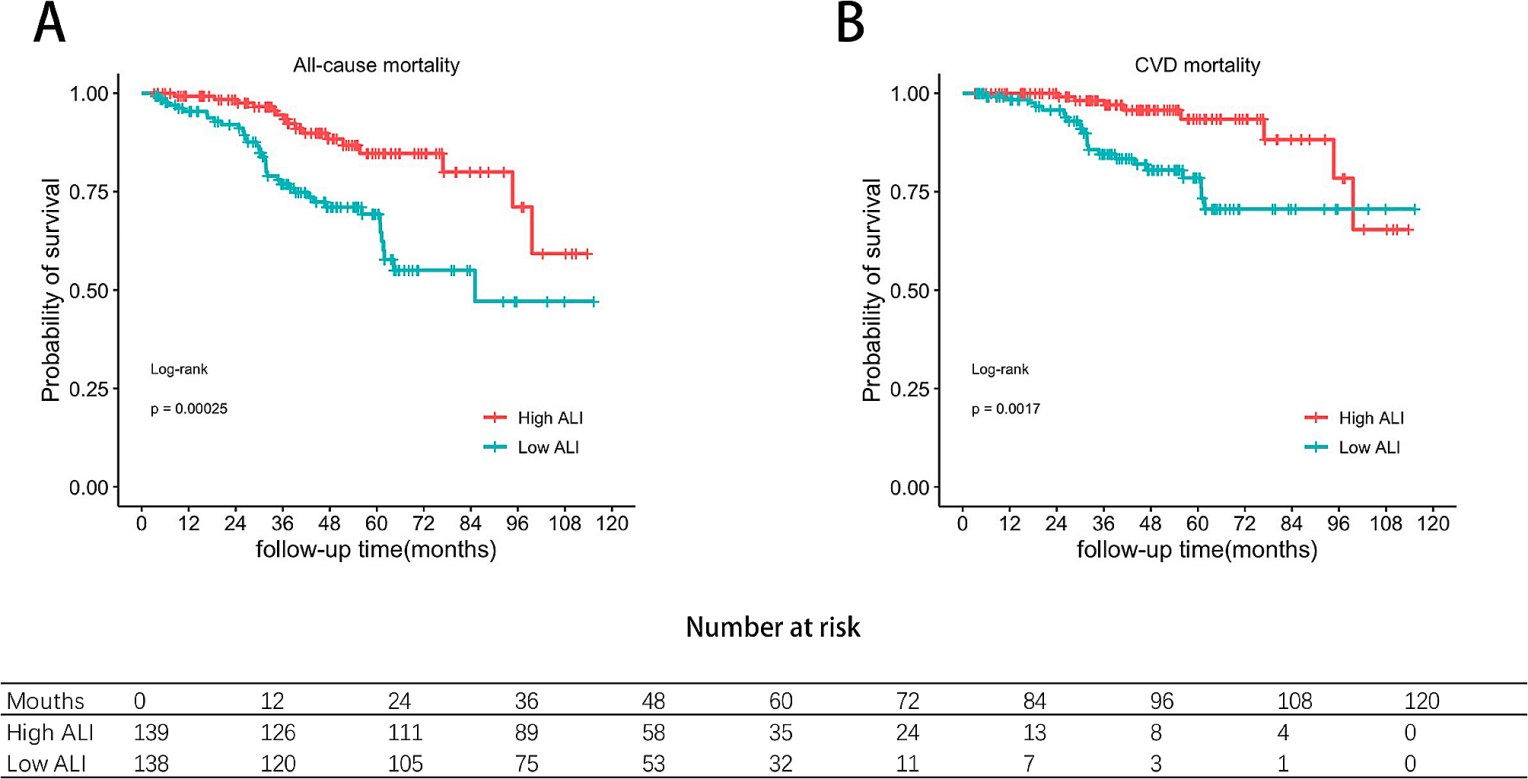
Comparison of the event-free survival rates for all-cause mortality (**A**) and CVD mortality (**B**) between the low-ALI group and the high-ALI group. ALI advanced lung cancer inflammation index, CVD cardiovascular disease

**Fig. 3:**
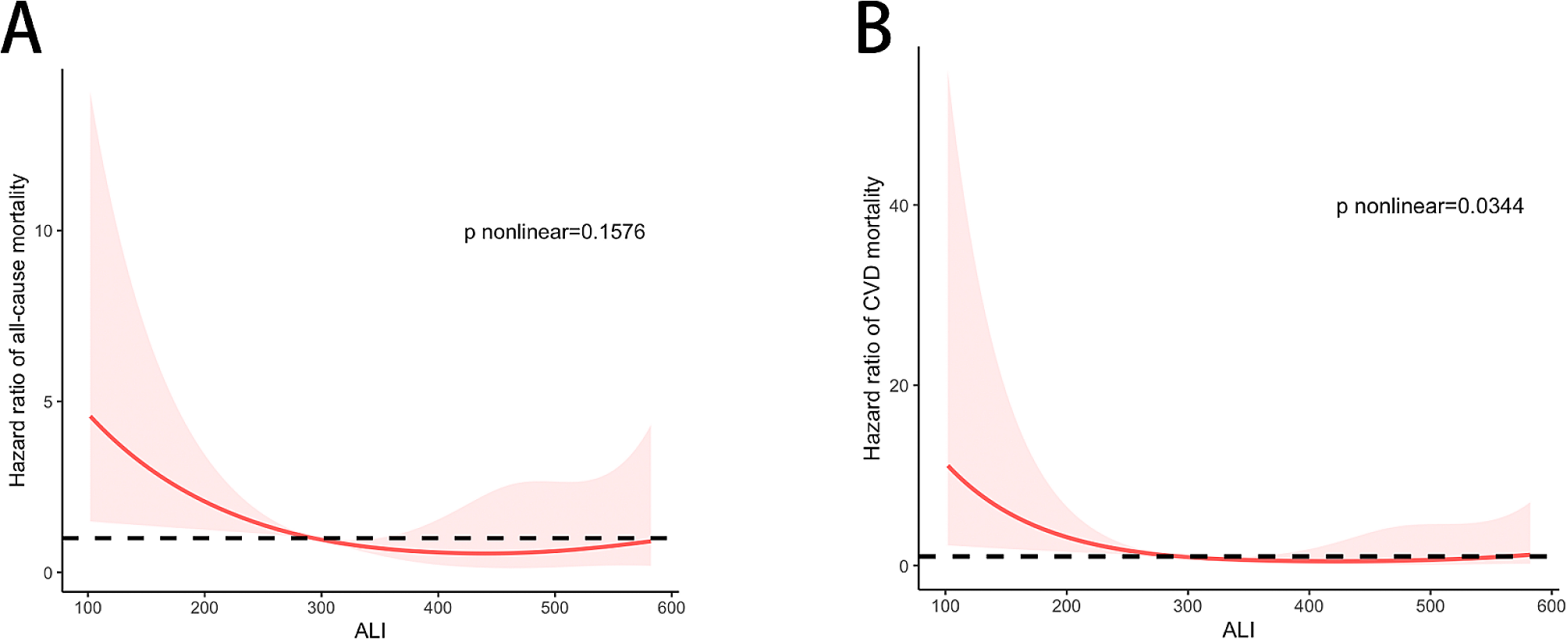
Multivariable-adjusted restricted cubic spline plots of HRs for all-cause mortality (**A**) and CVD mortality (**B**) according to the ALI (as a continuous measure). The solid line represents the HR, and the shaded area indicates the 95% confidence intervals. The horizontal dotted line corresponds to the normal reference HR of 1.0. The overall median value of ALI (ALI = 293.07) was chosen as the reference. The multivariable-adjusted model was adjusted for Model 4 (see Table 2). HR hazard ratio, CVD cardiovascular disease, ALI advanced lung cancer inflammation index

During a median follow-up of 40.5 months, 55 participants died. Of 55 deaths, 31 (56.36%) deaths were due to CVD, 6 (10.91%) deaths due to infection, 9 (16.36%) deaths due to multiple organ failure, 2 (3.63%) deaths due to respiratory failure, and 7 (12.73%) deaths due to other reasons. Kaplan–Meier curves revealed that the cumulative and cardiovascular cumulative survival rates of participants in the low-ALI group decreased significantly (all *P* < 0.001; Fig. 2). The 1-, 3-, and 5-year estimated cumulative survival rates in the low-ALI group were 95.4%, 77.0% and 67.0%, respectively. The 1-, 3-, and 5-year estimated cumulative survival rates in the high-ALI group were 99.2%, 93.4% and 84.7%, respectively (Fig. 2A). The 1-, 3-, and 5-year estimated cardiovascular cumulative survival rates in the low-ALI group were 98.4%, 84.6% and 75.9%, respectively. The 1-, 3-, and 5-year estimated cardiovascular cumulative survival rates in the high-ALI group were 100.0%, 97.1% and 93.4%, respectively (Fig. 2B). After multivariable adjustment in Model 4, patients in the low-ALI group had a 1.944-fold (95% CI 1.068–3.540, *P* = 0.030) greater risk of all-cause mortality and a 2.672-fold (95% CI 1.188–6.009, *P* = 0.017) greater risk of CVD mortality than patients in the high-ALI group did (Table 2). After adjusting for other confounders, we found a linear association between the ALI and the risk of all-cause mortality (P nonlinear = 0.1576). The risk of all-cause mortality increased with a decreasing ALI (Fig. 3A). However, there was a nonlinear relationship between the ALI and the risk of CVD mortality (nonlinear *P* = 0.0344; Fig. 3B).

**Table 2:** Association of the ALI with all-cause and CVD mortality in PD patients

Characteristic	Low ALI			High ALI	
	HR (95% CI)	*P* value		HR (95% CI)	*P* value
All-cause mortality					
Unadjusted	2.834 (1.581, 5.078)	< 0.001		1.000 (reference)	/
Model 1^a^	2.093 (1.153, 3.800)	0.015		1.000 (reference)	/
Model 2^b^	1.977 (1.092, 3.579)	0.024		1.000 (reference)	/
Model 3^c^	1.927 (1.060, 3.503)	0.031		1.000 (reference)	/
Model 4^d^	1.944 (1.068, 3.540)	0.030		1.000 (reference)	/
CVD mortality					
Unadjusted	3.378 (1.508, 7.568)	0.003		1.000 (reference)	/
Model 1^a^	2.776 (1.217, 6.333)	0.015		1.000 (reference)	/
Model 2^b^	2.667 (1.182, 6.018)	0.018		1.000 (reference)	/
Model 3^c^	3.203 (1.400, 7.331)	0.006		1.000 (reference)	/
Model 4^d^	2.672 (1.188, 6.009)	0.017		1.000 (reference)	/

^a^Model 1 was only adjusted for age

^b^Model 2 was further adjusted for sex, diabetes mellitus status, CVD status, smoking status, alcohol consumption status, systolic blood pressure, diastolic blood pressure and BMI

^c^Model 3 was further adjusted for medications (ACEIs or ARBs, β-blockers and calcium channel blockers)

^d^Model 4 was further adjusted for laboratory markers (Hb, ALB, hs-CRP, SUA, fasting blood glucose, TC, TG, HDL-C, and LDL-C) and 4-h D/Pcr (low transport: <0.50; low-average transport: 0.50–0.64; high-average transport: 0.65–0.81; high transport: >0.81)

ALI advanced lung cancer inflammation index, CVD cardiovascular disease, PD peritoneal dialysis, CI confidence interval, HR hazard ratio; BMI body mass index; ACEIs angiotensin-converting enzyme inhibitors; ARBs angiotensin receptor blockers; Hb hemoglobin; ALB albumin; hs-CRP high sensitivity C-reactive protein; SUA serum uric acid; TC total cholesterol; TG triglyceride; HDL-C high-density lipoprotein cholesterol; LDL-C low-density lipoprotein cholesterol; 4-h D/Pcr 4-h dialysate-to-plasma ratio of creatinine

### Prognostic value of the ALI for all-cause and CVD mortality outcomes

**Fig. 4:**
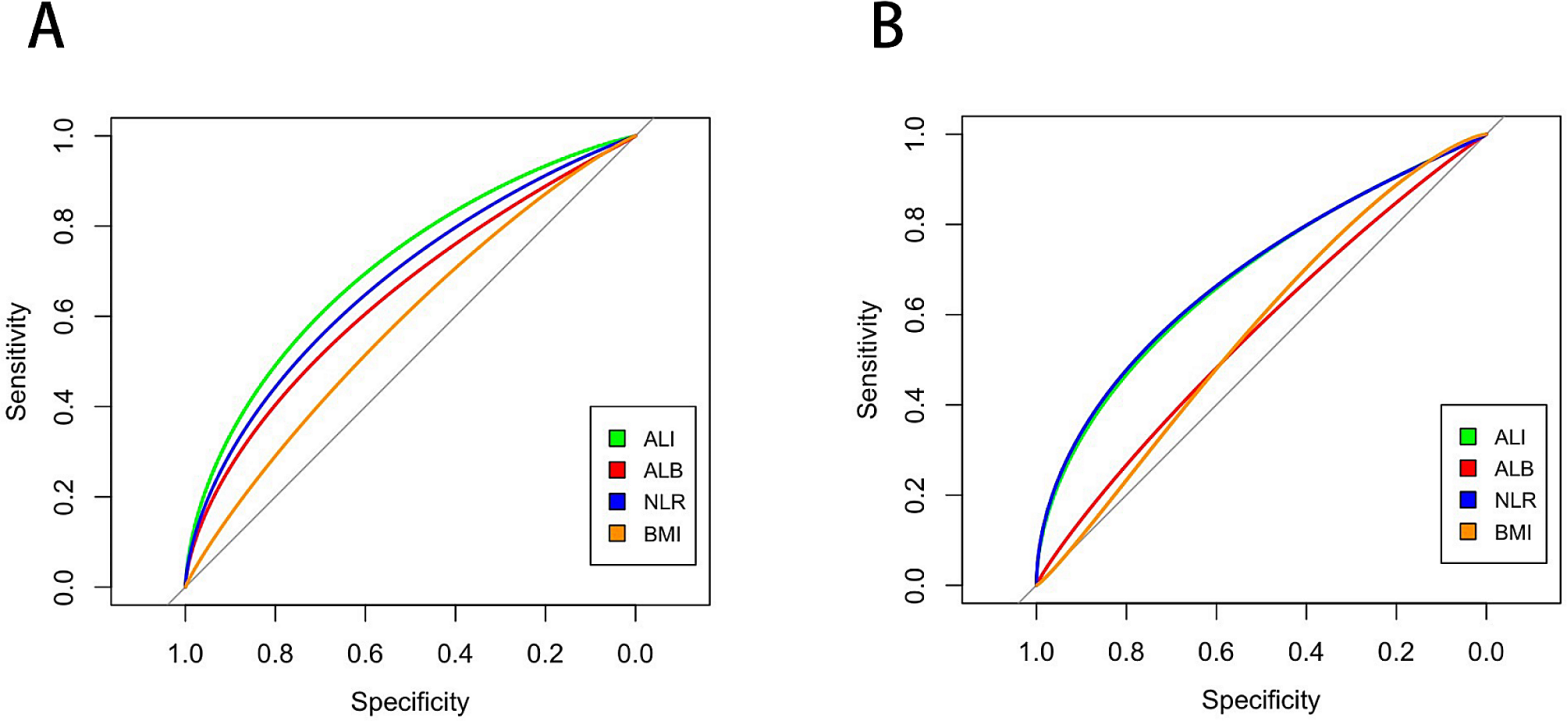
ROC curves for the associations of the ALI, ALB, NLR, and BMI with all-cause mortality (**A**) and CVD mortality (**B**). ROC receiver operating characteristic, ALI advanced lung cancer inflammation index, ALB albumin, NLR neutrophil-to-lymphocyte ratio, BMI body mass index, CVD cardiovascular disease

The ROC curves showed that the AUCs for the baseline ALI, ALB, NLR and BMI for the prediction of all-cause mortality were 0.708 (95% CI 0.630–0.786, *P* < 0.001), 0.644 (95% CI 0.556–0.726, *P* < 0.001), 0.675 (95% CI 0.596–0.754, *P* < 0.001) and 0.581 (95% CI 0.469–0.659, *P* = 0.069), respectively. When cardiovascular mortality was used as an endpoint, the AUC for the ALI was 0.683 (95% CI 0.573–0.783, *P* = 0.001), the AUC for the ALB was 0.558 (95% CI 0.445–0.651, *P* = 0.302), the AUC for the NLR was 0.687 (95% CI 0.576–0.784, *P* < 0.001) and the AUC for the BMI was 0.563 (95% CI 0.464–0.660, *P* = 0.257). The ALI was superior to the ALB, NLR, and BMI in predicting all-cause mortality. However, the NLR had a better ability to predict cardiovascular mortality (Fig. 4; Table 3).

**Table 3:** AUCs of the ALI, ALB, NLR, and BMI for all-cause and CVD mortality

	AUC (95% CI)	*P* Value	Cut-off value	Youden Index	Sensitivity (%)	Specificity (%)
All-cause mortality						
ALI	0.708 (0.630, 0.786)	< 0.001	≤ 224.4993	0.361	56.4	79.7
ALB	0.644 (0.556, 0.726)	< 0.001	≤ 28.815	0.288	49.1	79.7
NLR	0.675 (0.596, 0.754)	< 0.001	> 3.0842	0.297	54.5	75.2
BMI	0.581 (0.496, 0.659)	0.069	≤ 24.025	0.173	72.7	44.6
CVD mortality						
ALI	0.683 (0.573, 0.783)	0.001	≤ 222.1859	0.393	61.3	78.0
ALB	0.558 (0.445, 0.651)	0.302	≤ 29.125	0.192	45.2	74.0
NLR	0.687 (0.576, 0.784)	< 0.001	> 3.0886	0.349	61.3	73.6
BMI	0.563 (0.464, 0.660)	0.257	≤ 25.085	0.180	83.9	34.1

ALI advanced lung cancer inflammation index, ALB albumin, NLR neutrophil-to-lymphocyte ratio, BMI body mass index, CVD cardiovascular disease, AUC area under the curve, CI confidence interval

## Discussion

This retrospective study revealed that a lower ALI was independently associated with all-cause (linear association) and CVD (nonlinear association) mortality. In addition, the ALI was shown to be superior to individual parameters for predicting all-cause mortality.

ALB is a highly water-soluble protein synthesized by the liver, and its synthesis as a negative acute-phase protein is influenced by inflammation and blood volume status [12, 13]. Therefore, the ALB is not a sensitive indicator for assessing malnutrition in chronic kidney disease (CKD) patients [14]. The BMI is an objective index that can be easily obtained in the clinic to evaluate systemic nutrition, but it is also affected by fat mass and hydration status [15, 16]. A special state of chronic inflammatory immunosuppression exists in CKD patients, and some scholars have named this a CKD-associated immune dysfunction that is manifested by abnormal activation of the innate immune system (neutrophil involvement) and dysfunction of the adaptive immune system (lymphocyte involvement) [17]. The NLR was calculated as the peripheral blood neutrophil count divided by the lymphocyte count. A higher NLR may reflect an imbalance between the two immune responses in CKD patients [18]. The ALI is a combination of the ALB, BMI and NLR and has been proven to be related to the prognosis of non-small cell lung cancer, gastrointestinal tumours, diffuse large B-cell lymphoma, head and neck tumours, etc [7, 19–21]. A recent study showed that the ALI was associated with long-term all-cause mortality in hypertensive patients and was used as a comprehensive indicator of nutrition status and inflammation [22]. Another study revealed that the ALI was a valid predictor of all-cause and cardiovascular mortality in elderly patients with heart failure. When comparing the value of the geriatric nutritional risk index (GNRI) and the ALI in evaluating long-term mortality, the results indicated that both indices have comparable predictive value for all-cause and cardiovascular mortality at 2 years [10]. A large cohort study enrolling 9727 cancer patients compared the associations of different inflammatory markers with sarcopenia in cancer patients. The results showed that the ALI was superior to the NLR, prognostic nutritional index (PNI), systemic immunoinflammatory index (SII) and platelet-to-lymphocyte ratio (PLR) for predicting and differentiating sarcopenia. Patients with a low ALI and severe malnutrition had a 2.262-fold increased risk of mortality [23]. Like many chronic diseases, CKD patients exhibit a prolonged state of low-grade inflammation. Persistent inflammation accelerates PEW by promoting systemic protein catabolism, inducing anorexia, reducing voluntary activity and promoting insulin resistance [24, 25]. Our study also confirmed the close relationship between a low ALI and adverse outcomes in PD patients. Multivariate Cox regression analyses revealed that a lower ALI is an independent risk factor for all-cause mortality and cardiovascular mortality in PD patients. Survival curves showed that the risk of all-cause and cardiovascular mortality was significantly greater in the low-ALI group than in the high-ALI group. In this study, we further compared the ability of the ALI, the ALB, the NLR, and the BMI to predict mortality in PD patients. The ROC curve showed that the ALI had the highest efficiency in predicting all-cause mortality, indicating that the ALI might be a more comprehensive index for predicting all-cause mortality in PD patients than a single indicator.

Hs-CRP is a recognized biomarker of inflammation and plays an important role in the occurrence and development of atherosclerosis. Many studies have proven that an increase in hs-CRP at a single time point is an independent predictor of all-cause and cardiovascular mortality in dialysis patients [26–28]. However, Li et al. reached a different conclusion. They found that higher longitudinal hs-CRP levels over time rather than higher baseline levels were predictive of both all-cause and CVD mortality [29]. Consistent with the results of Li et al., we found that baseline hs-CRP was not associated with all-cause mortality or CVD mortality, even after adjustment for other parameters (Supplementary Table 1). There are many possible reasons for this result. One explanation may be that hs-CRP fluctuated significantly during the follow-up period in this study. Studies have shown that the correlation between higher CRP levels and poor prognosis in patients who have been followed up for a long time (2–3 years of follow-up) is not as good as that in patients who have been followed up for a short time (1 year of follow-up) [30]. Another possible reason could be related to our small sample size, which may have biased the results. We further plotted an ROC curve to evaluate the predictive value of baseline hs-CRP for all-cause mortality and cardiovascular mortality in PD patients. Our results suggested that baseline hs-CRP levels have limited value in predicting the adverse outcomes in PD patients (Supplementary Fig. 1). The baseline ALI was better at predicting all-cause and CVD mortality than was the hs-CRP, which implied that the ALI might be a potential marker for evaluating the prognosis of PD patients.

In this study, the proportion of patients with diabetes mellitus and CVD was greater in the low-ALI group. A previous prospective study demonstrated that dialysis patients with comorbidities were more likely to be malnourished, and the relative risk of malnutrition in patients with comorbidities was 3.9 times greater than that in patients without comorbidities [31]. In another study of 683 CKD patients in China, Dai et al. reported that the incidence of malnutrition was significantly greater in CKD patients with diabetes mellitus than in CKD patients without diabetes mellitus [32]. We also found that PD patients with a lower ALI had greater peritoneal transport. Previous studies have suggested that the incidence of malnutrition may be significantly greater in patients with high peritoneal transport. Liu et al. followed 283 continuous ambulatory peritoneal dialysis (CAPD) patients for 1 year and reported that higher peritoneal transport status at baseline was strongly associated with poor short-term nutritional status in CAPD patients [33]. The mechanism may include a high level of inflammation as well as increased glucose absorption from the dialysate in high peritoneal transport states, leading to a loss of appetite. A recent study demonstrated that a low PNI was independently associated with high peritoneal transit status after adjusting for relevant influencing factors (OR = 3.42, 95% CI 1.43 to 8.15) [34]. In addition, our results also showed that patients in the low-ALI group had lower Hb levels. One study divided HD patients into the PEW group and the non-PEW groups according to the malnutrition inflammation score (MIS); patients in the PEW group responded poorly to treatment with erythropoietin and tended to be more anaemic in combination with malnutrition [35, 36].

This study has several limitations. First, this was a single-centre, retrospective study with a small sample size, which might have resulted in selection bias and the follow-up time was insufficient. Second, we collected data only for the initial period of dialysis and did not dynamically monitor the data during the follow-up period. Third, although we included common risk factors such as age, sex and comorbidities in our multivariate model, there may have been other unmeasured confounders that affected the results. Finally, our study lacked the comparison between ALI and common clinical prediction tools. Therefore, we plan to continue expanding the sample size and include other tools for assessing PEW, such as subjective global assessment (SGA) and MIS, in the future. We will compare these indicators with the ALI in predicting the prognosis of PD patients.

In summary, the ALI is an inexpensive and easily accessible marker that can be used to assess the prognosis of PD patients. Prospective studies with large sample sizes and multiple centres are needed to further validate our results.

### Electronic supplementary material

Below is the link to the electronic supplementary material.

Supplementary Material 1

## Data Availability

The datasets used and analyzed during the current study available from the corresponding author on reasonable request.

## Abbreviations

ALI: Advanced lung cancer inflammation index
PD: Peritoneal dialysis
PEW: Protein-energy wasting
HD: Haemodialysis
CVD: Cardiovascular disease
BMI: Body mass index
WBC: White blood cell
Hb: Hemoglobin
hs-CRP: High sensitivity C-reactive protein
ALB: Albumin
Scr: Serum creatinine
SUA: Serum uric acid
TC: Total cholesterol
TG: Triglyceride
HDL-C: High-density lipoprotein cholesterol
LDL-C: Low-density lipoprotein cholesterol
Kt/V: Total urea clearance index
CCR: Creatinine clearance rate
4-h D/Pcr: 4-h dialysate-to-plasma ratio of creatinine
eGFR: Estimated glomerular filtration rate
NLR: Neutrophil-to-lymphocyte ratio
ACEIs: Angiotensin-converting enzyme inhibitors
ARBs: Angiotensin receptor blockers
ROC: Receiver operating characteristic
AUC: Area under the curve
RASIs: Renin-angiotensin system inhibitors
HR: Hazard ratio
CI: Confidence interval
CKD: Chronic kidney disease
GNRI: Geriatric nutritional risk index
PNI: Prognostic nutritional index
SII: Systemic immunoinflammatory index
PLR: Platelet-to-lymphocyte ratio
CAPD: Continuous ambulatory peritoneal dialysis
SGA: Subjective Global Assessment
MIS: Malnutrition inflammation score
