# Supplementary material for: The advanced lung cancer inflammation index is associated with mortality in peritoneal dialysis patients
Source: BMC Nephrol. 2024 Jun 25;25:208. doi: 10.1186/s12882-024-03645-4 (PMC11201339; doi:10.1186/s12882-024-03645-4)
Supplement: Supplementary file 1 — Supplementary Material 1 [file 12882_2024_3645_MOESM1_ESM.docx]

**
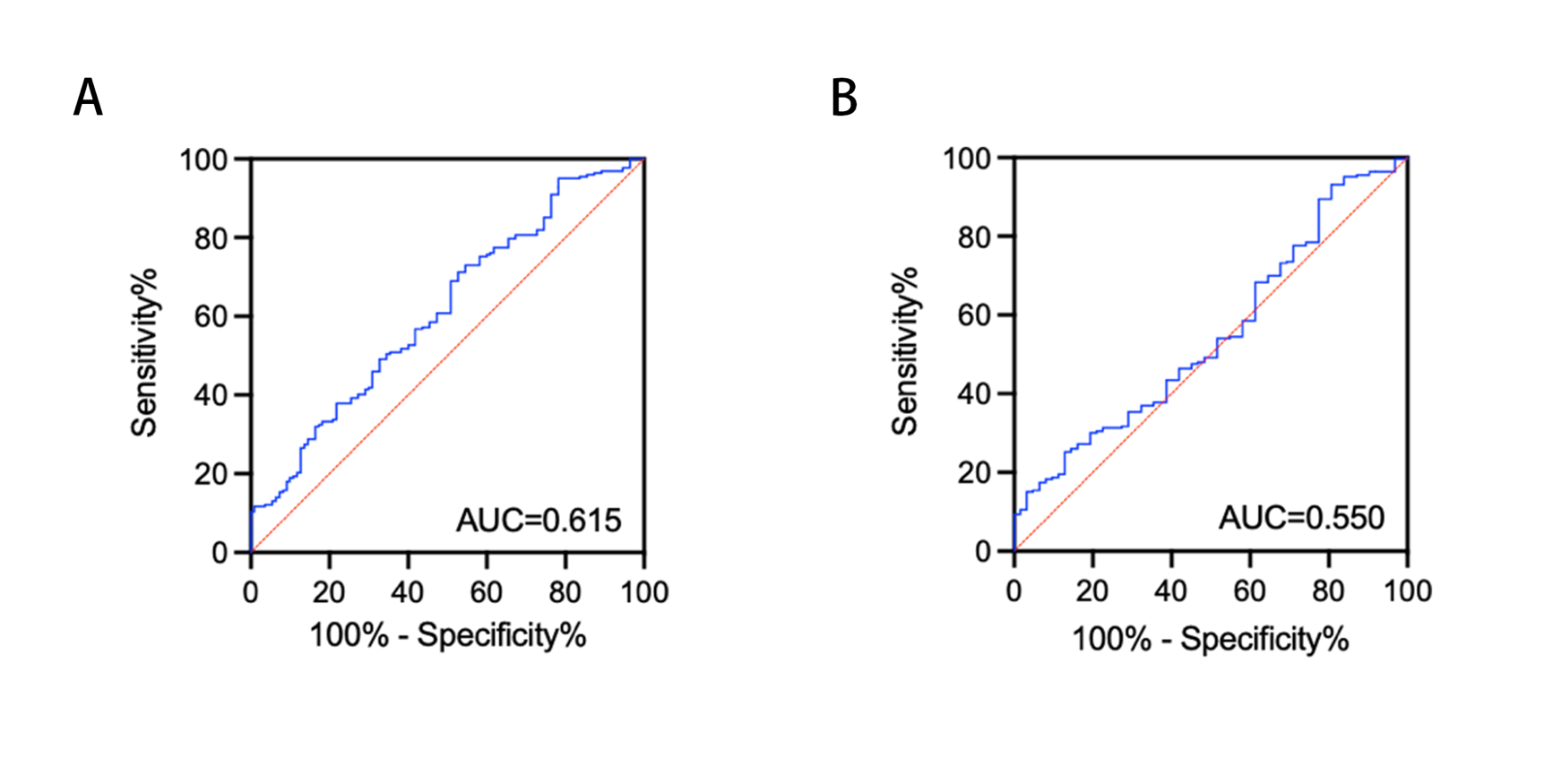
**

**Supplementary Figure 1** ROC curves of hs-CRP to predict all-cause mortality (A) and cardiovascular mortality (B) in PD patients. hs-CRP high sensitivity C-reactive protein; AUC area under the curve; ROC Receiver operating characteristic; PD peritoneal dialysis

**Supplementary Table 1** Association of the hs-CRP with all-cause and CVD mortality in PD patients

|  | Unadjusted |  |  | Model 1^a^ |  |  | Model 2^b^ |  |  | Model 3^c^ |  |
| --- | --- | --- | --- | --- | --- | --- | --- | --- | --- | --- | --- |
|  | HR (95% CI) | P value |  | HR (95% CI) | P value |  | HR (95% CI) | P value |  | HR (95% CI) | P value |
| All-cause mortality |  |  |  |  |  |  |  |  |  |  |  |
| Hs-CRP, per 1 mg/L | 1.035 (1.020, 1.050) | <0.001 |  | 1.021 (1.005, 1.038) | 0.009 |  | 1.021 (1.004, 1.038) | 0.017 |  | 1.017 (0.998, 1.037) | 0.078 |
| Hs-CRP<2.09 mg/L | 1 (reference) |  |  | 1 (reference) |  |  | 1 (reference) |  |  | 1 (reference) |  |
| Hs-CR≥2.09 mg/L | 1.766 (1.028, 3.032) | 0.039 |  | 1.288 (0.715, 2.323) | 0.399 |  | 1.120 (0.609, 2.060) | 0.715 |  | 1.262 (0.649, 2.454) | 0.492 |
| CVD mortality |  |  |  |  |  |  |  |  |  |  |  |
| Hs-CRP, per 1 mg/L | 1.032 (1.010, 1.053) | 0.004 |  | 1.020 (0.997, 1.043) | 0.088 |  | 1.020 (0.997, 1.044) | 0.087 |  | 1.025 (0.998, 1.052) | 0.069 |
| Hs-CRP<2.09 mg/L | 1 (reference) |  |  | 1 (reference) |  |  | 1 (reference) |  |  | 1 (reference) |  |
| Hs-CR≥2.09 mg/L | 1.121 (0.553, 2.272) | 0.752 |  | 0.851(0.396, 1.851) | 0.680 |  | 0.753 (0.339, 1.673) | 0.486 |  | 0.849 (0.355, 2.031) | 0.714 |

Model 1^a^ was adjusted for age, sex, diabetes mellitus status, CVD status, smoking status, alcohol consumption status, systolic blood pressure, diastolic blood pressure and BMI

Model 2^b^ was further adjusted for medications (ACEIs or ARBs, β-blockers and calcium channel blockers)

Model 3^c^ was further adjusted for laboratory markers (Hb, ALB, SUA, fasting blood glucose, TC, TG, HDL-C, and LDL-C).

hs-CRP high sensitivity C-reactive protein; CVD cardiovascular disease, PD peritoneal dialysis, CI confidence interval, HR hazard ratio; BMI body mass index; ACEIs angiotensin-converting enzyme inhibitors; ARBs angiotensin receptor blockers; Hb hemoglobin; ALB albumin; SUA serum uric acid; TC total cholesterol; TG triglyceride; HDL-C high-density lipoprotein cholesterol; LDL-C low-density lipoprotein cholesterol
